# Dysregulation of Mitochondrial Quality Control Processes Contribute to Sarcopenia in a Mouse Model of Premature Aging

**DOI:** 10.1371/journal.pone.0069327

**Published:** 2013-07-23

**Authors:** Anna-Maria Joseph, Peter J. Adhihetty, Nicholas R. Wawrzyniak, Stephanie E. Wohlgemuth, Anna Picca, Gregory C. Kujoth, Tomas A. Prolla, Christiaan Leeuwenburgh

**Affiliations:** 1 Division of Biology of Aging, Department of Aging and Geriatric Research, University of Florida, Gainesville, Florida, United States of America; 2 Department of Applied Physiology and Kinesiology, University of Florida, Gainesville, Florida, United States of America; 3 Department of Molecular Genetics and Microbiology, University of Florida, Gainesville, Florida, United States of America; 4 Department of Genetics, University of Wisconsin, Madison, Wisconsin, United States of America; Pennington Biomed Research Center, United States of America

## Abstract

Mitochondrial DNA (mtDNA) mutations lead to decrements in mitochondrial function and accelerated rates of these mutations has been linked to skeletal muscle loss (sarcopenia). The purpose of this study was to investigate the effect of mtDNA mutations on mitochondrial quality control processes in skeletal muscle from animals (young; 3–6 months and older; 8–15 months) expressing a proofreading-deficient version of mtDNA polymerase gamma (PolG). This progeroid aging model exhibits elevated mtDNA mutation rates, mitochondrial dysfunction, and a premature aging phenotype that includes sarcopenia. We found increased expression of the mitochondrial biogenesis regulator peroxisome proliferator-activated receptor gamma coactivator-1α (PGC-1α) and its target proteins, nuclear respiratory factor 1 (NRF-1) and mitochondrial transcription factor A (Tfam) in PolG animals compared to wild-type (WT) (P<0.05). Muscle from older PolG animals displayed higher mitochondrial fission protein 1 (Fis1) concurrent with greater induction of autophagy, as indicated by changes in Atg5 and p62 protein content (P<0.05). Additionally, levels of the Tom22 import protein were higher in PolG animals when compared to WT (P<0.05). In contrast, muscle from normally-aged animals exhibited a distinctly different expression profile compared to PolG animals. Older WT animals appeared to have higher fusion (greater Mfn1/Mfn2, and lower Fis1) and lower autophagy (Beclin-1 and p62) compared to young WT suggesting that autophagy is impaired in aging muscle. In conclusion, muscle from mtDNA mutator mice display higher mitochondrial fission and autophagy levels that likely contribute to the sarcopenic phenotype observed in premature aging and this differs from the response observed in normally-aged muscle.

## Introduction

Mitochondria play a fundamental role in several key cellular processes which include but are not limited to energy production, calcium signaling, reactive oxygen species (ROS) generation, and cell death (apoptosis) [1]. Dysregulation in any one of these processes can influence the quantity and/or quality of mitochondria, and potentially trigger a cascade of detrimental events within the cell. This has been proposed to be the case in aging skeletal muscle, where over time chronic elevations in oxidative stress can cause cumulative and irreversible damage to mitochondrial proteins, lipids, and nucleic acids. In particular, oxidative stress-induced damage to mtDNA impairs mitochondrial function which can lead to further increases in ROS production and exacerbate the intracellular ROS-induced damage. This phenomenon known as the mitochondrial theory of aging, postulates that this will eventually lead to insurmountable damage and the activation of cell death pathways, that ultimately contribute to muscle wasting and the functional decline in muscle termed sarcopenia [2]–[4].

Increased levels of mtDNA point mutations and deletions are documented in a wide range of aged tissues in both humans and animals, and have been linked to a number of pathological conditions [5]–[8]. Additional support for the role of mtDNA mutations and mitochondrial dysfunction in aging is provided by the mtDNA mutator mouse (designated PolG) that expresses a proofreading-deficient version of mitochondrial polymerase gamma (D257A). This proofreading defect causes rapid accumulation of mtDNA point mutations and deletions with age predisposing these animals to a premature aging phenotype and a significantly reduced lifespan when compared to wild-type animals [9]–[11]. Specifically, animals display premature signs of aging that begin as early as 8 months of age and include greying, alopecia, kyphosis, age-related hearing loss (presbycusis), osteoporosis, and sarcopenia [9], [10]. Our previous work characterizing the sarcopenic phenotype in these mice revealed marked atrophy of ∼20% in gastrocnemius muscle of older PolG animals when compared to control animals. This atrophy in 11-month PolG mice represents a level of sarcopenia typically observed in 30-month normally-aged WT animals [12]. Loss of muscle mass observed in PolG mice is tightly associated with reduced electron transport chain (ETC) complexes, impaired mitochondrial bioenergetics, and the induction of apoptosis. However, these changes occur in the absence of the higher ROS production evident in chronologically (normal) aging WT muscle which may implicate alternative damage-inducing stressors in muscle wasting within PolG mice [9], [10], [12], [13]. It is important to note that while the use of this premature aging model to establish a causal relationship between mtDNA mutations and mammalian aging remains under question, it certainly provides a useful tool to study the effects of increased mtDNA mutations/mitochondrial dysfunction in muscle, a common feature of normally-aging tissue.

Mitochondria have many defense pathways to combat excessive damage and maintain quality control, and this is particularly important for post-mitotic tissue such as skeletal muscle. Mitochondria are not static organelles but instead quite dynamic, continuously being reorganized and/or recycled via fusion and fission processes. Biogenesis of new organelles is regulated by the peroxisome proliferator-activated receptor gamma coactivator 1-alpha (PGC-1α), as well as a number of fusion and fission proteins. The latter proteins not only dictate the morphology of this complex interconnected network but are also important for energy metabolism, redox signaling, and cell death. Moreover, mitochondrial fusion facilitates the mixing of mtDNA from one organelle to another and prevents a high concentration of mutant mtDNA from accumulating [14]. This suggests that mitochondrial fusion is an important component in the maintenance of mtDNA integrity [15]. Additionally, fission segregates functionally damaged organelles and targets them for removal through autophagy [15], [16]. This catabolic process degrades damaged organelles and/or proteins through sequestration into lysosomal machinery preventing the accumulation of functionally impaired components and damage to the cell [17]. The failure of autophagy to degrade and eliminate damaged organelles/proteins has been implicated in the functional decline of tissues that is observed with aging [18], [19]. Thus, skeletal muscle mass is tightly regulated by mitochondrial quality control processes and slight perturbations in these pathways can lead to greater myocellular damage and atrophy.

The purpose of this study was to investigate whether mitochondrial quality control processes are altered in an animal model of progeroid aging that exhibits high mtDNA mutations, mitochondrial dysfunction, and most importantly a sarcopenic phenotype that is commonly observed in normally-aging mice. These animals were not used as a model of chronological aging but as a tool to study and understand the specific effects of mtDNA mutations/mitochondrial dysfunction on muscle wasting. MtDNA mutator mice (PolG) begin to display muscle wasting as early as 8 months of age [9] and therefore animals were divided into two groups, young (3–6 months) and older (8–15 months), the latter representing the animals with higher mtDNA mutation rates and premature sarcopenia. Additionally, we also assessed the effect of normal aging (WT) on these mitochondrial quality control pathways and compared them to the PolG animals. Based on our previous work, we hypothesized that similar to chronological aging, muscle from PolG animals would exhibit impaired mitochondrial quality control processes resulting from reduced mitochondrial fission and/or higher fusion and lower autophagy degradation leading to greater mitochondrial dysfunction and premature sarcopenia.

## Materials and Methods

### Animals

All animal research was conducted in accordance with the regulatory policies of the Institutional Animal Care and Use Committees of the University of Florida (CL approved protocol #D420) and all efforts were made to minimize suffering to the animals. Transgenic mice expressing a proofreading-deficient version of the mitochondrial DNA polymerase gamma (D257A) gene referred to as PolG were generated as previously described [9]. These mice have higher rates of mtDNA mutagenesis and display a premature aging phenotype. Animals were housed in quarantines in a climate- and light-controlled environment. Following one week of acclimation, animals were sacrificed by way of rapid cervical dislocation. Both male and female animals were combined and used in the study as no previous gender differences were observed based on the measurements being performed in this study [20]. WT and PolG mice (C57B1/6J background) were sacrificed at 2 age groups (young: 3–6 months and older: 8–15 months). The older age group was chosen because muscle mass loss in PolG animals begins at ∼ 8 months of age [9]. Once removed, tissues were immediately placed in liquid N_2_ and stored for further analysis.

### Cytochrome c oxidase (COX) Enzyme Activity

Cytochrome c oxidase (COX) activity was measured as previously described [21]. Briefly, powdered tissues were diluted in a buffer (0.1 M KH_2_PO4+2 mM EDTA, pH 7.2) and sonicated (3×5 s) on ice. Following a brief spin, the supernatant was removed and enzyme activity determined by the maximal oxidation rate of completely reduced cytochrome *c*, evaluated as a change in absorbance at 550 nm using a multi-detection microplate reader (Synergy HT, Biotek Instruments, Winooski, VT).

### Mitochondrial DNA Content

Total DNA was prepared from 2 mg of muscle using NucleoSpin Tissue Kit (Macherey Nagel) and stored at −20°C. Real Time PCR (qRT-PCR) was used to measure mtDNA content as previously described [22]. Briefly, primers for mouse mitochondrial DNA were For: 5′-AAT CTA CCA TCC TCC GTG AAA CC-3′; Rev: 5′GCC CGG AGC GAG AAG AG-3′ and for mouse nuclear beta-actin gene were beta-actinFor: 5′-AGC CAT GTA CGT AGC CAT CCA-3′; beta-actinRev: 5′GCC CGG AGC GAG AAG AG-3′. Samples were analyzed in triplicate in a final volume of 25 µl consisting of iTaq SYBR Green Supermix PCR 1× Master Mix (Bio-Rad Laboratories Inc., Hercules, CA, USA), 0.2 µM forward and reverse primers and DNA template. Reactions were performed with an ABI PRISM 300 Sequence Detection System (Applied biosystems, Foster City, CA, USA). Samples were denatured for 10 min at 95°C, followed by 40 cycles of amplification, each consisting of denaturation at 95°C for 15 sec, and annealing and extension at 60°C for 1 min. Relative mtDNA content was normalized to β-actin and quantified using the Pfaffl mathematical model [23].

### Immunoblotting

Frozen quadriceps muscles were pulverized and protein extracts prepared as previously described [21]. Muscle extracts (50 µg/lane) were separated by 4–15% SDS-PAGE and subsequently electroblotted to nitrocellulose membranes. After transfer, membranes were blocked by incubating for 1 hr at room temperature in Starting Block T20 Blocking Buffer (Thermo Scientific) and incubated overnight at 4°C in blocking buffer at a dilution of 1∶500 for Phosphorylated AMPKα and Total AMPKα (Cell Signaling, 2531 and 2532, respectively), 1∶500 for PGC-1α (Calbiochem, 516557), 1∶500 for NRF-1 (Santa Cruz, Sc-19050), 1∶1000 for Tfam (Calbiochem, DR-1071),1∶500 for Mfn1 (Santa Cruz, Sc-50330) and Mfn2 (Sigma, M6444), 1∶1000 for Opa1 and DRP1 (BD Transduction Laboratories, 612606 and 611112), 1∶500 for Fis1 (Alexis Biochemicals, ALX-210-907-R100), 1∶1000 for mtHsp70 and cHsp70 (Assay Designs, ADI-SPS-825 and ADI-SPS-810), 1∶500 for Tom22 (Sigma, T6319), 1∶1000 for Beclin-1 (Cell Signaling, 3738), 1∶1000 for LC3 (Cell Signaling, 2775), 1∶1000 for p62 (Sigma, P0067), 1∶500 for Atg5 (Cell Signaling, 2630), 1∶500 for ULK1 (Cell Signaling, 8054), 1∶5000 for actin (Sigma, A2066). The Atg5 antibody detects endogenous levels of total Atg5 but the band represents the Atg12-Atg5 conjugated form, as well as free Atg5. All commercially available antibodies used in this study have been well documented in previous papers published by our group [24], [25]. After overnight incubation, blots were washed in TBST (3×5 min), incubated at room temperature for 45 min with appropriate secondary antibodies, and washed again in TBST (3×5 min). Antibody binding was detected with the use of secondary antibodies conjugated to horseradish peroxidase and blots exposed using an enhanced chemiluminescence (ECL) detection kit (Santa Cruz Biotechnology, Santa Cruz, CA). Films were scanned and analyzed using the Kodak ID Imaging Software. All proteins were normalized for actin and Ponceau S staining.

### Statistical Analysis

All statistical analyses were carried out by two-way analysis of variance (ANOVA) to compare differences among multiple groups, followed by Bonferonni post-hoc analysis to specifically test individual differences between groups. Differences were considered statistically significant if *P*<0.05. Data are presented as mean ± standard error (SE).

## Results

### Mitochondrial Content and Enzyme Activity

**Figure 1:**
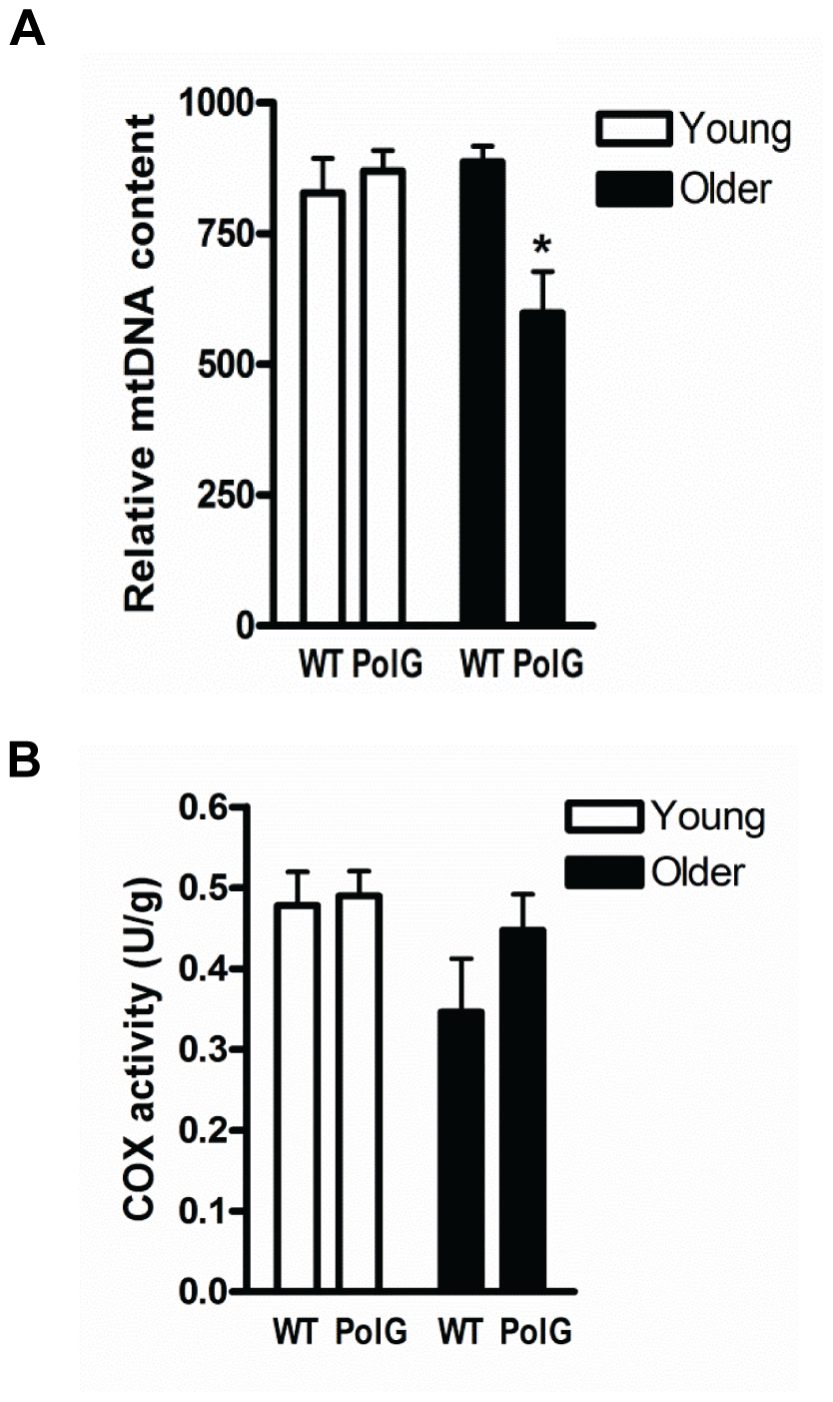
Mitochondrial DNA content and enzyme activity in muscle from PolG mice. *(A)* Mitochondrial DNA (mtDNA) was measured by Real Time-PCR (qT-PCR) in muscle from young (3–6 mo) and older (8–15 mo) WT and PolG mice. Relative mtDNA content was normalized to β-actin and a graphical representation of the summary data is shown (n = 7–9). (*B*) Cytochrome *c* oxidase (COX) activity was expressed as unit per gram of tissue (n = 7–9). Significance was set at P<0.05 and all data are represented as mean ± SE. *P<0.05 vs. age-matched WT.

We measured the relative levels of mtDNA, as well as cytochrome c oxidase (COX) activity as indicators of mitochondrial content and function [21], [22]. Our results showed reduced (P<0.05) mtDNA content in muscle from older mutator mice when compared to wild-type (WT; Fig. 1A). In contrast, there were no differences detected in COX activity among any of the groups even when normalized to total protein content (Fig. 1B).

### Mitochondrial Biogenesis Regulators are Upregulated in PolG Animals

**Figure 2:**
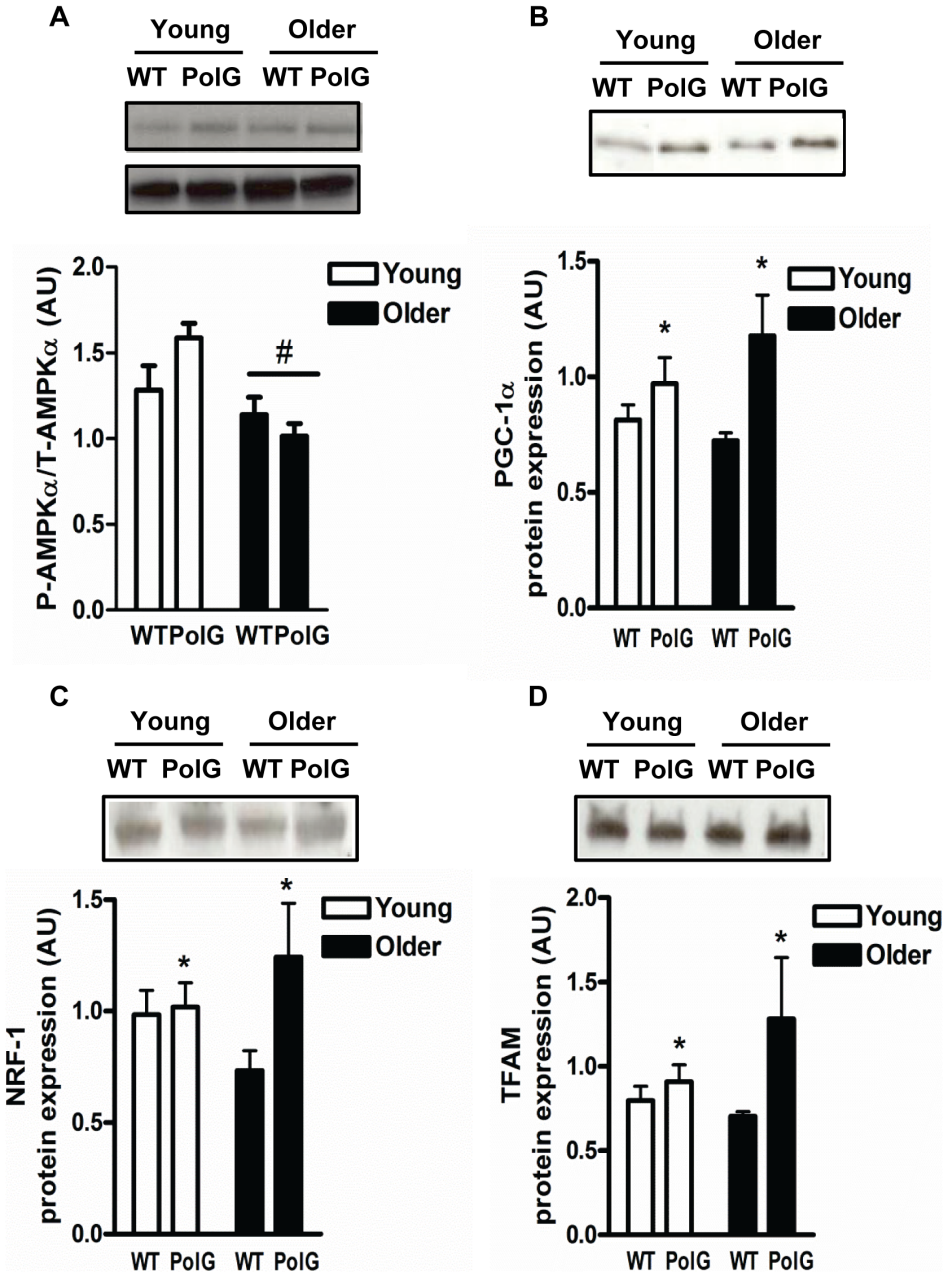
PolG mice display higher levels of mitochondrial regulatory proteins and transcription factors. AMPKα activation (*A*), PGC-1α (*B*), NRF-1 (*C*), and Tfam (*D*) were determined by Western Blotting in muscle from young (3–6 mo) and older (8–15 mo) WT and PolG animals. AMPKα activation is determined by phosphorylated AMPKα over total AMPKα. Representative blots are shown above with a graphical summary of the data below (n = 7–13). Significance was set at P<0.05 and all data are represented as mean ± SE. Data are expressed as arbitrary units (AU). #P<0.05 main effect of age. *P<0.05 main effect of genotype.

Mitochondrial biogenesis is required for the synthesis of new mitochondria and is also important for maintaining mitochondrial turnover. We found higher levels (P<0.05) of peroxisome proliferator-activated receptor gamma coactivator-1 alpha (PGC-1α; Fig. 2B), as well as its downstream target proteins, nuclear respiratory factor 1 (NRF-1; Fig. 2C) and mitochondrial transcription factor A (Tfam; Fig. 2D; P<0.05) in young and older PolG animals when compared to WT although these effects were more pronounced in the older. While there was an effect of age (P<0.05; Fig. 2A) on AMPK (5′ AMPK-activated protein kinase) activation, no age-induced effects were observed in any downstream mitochondrial biogenesis proteins measured.

### Altered Mitochondrial Morphology Proteins in mtDNA Mutator Mice

**Figure 3:**
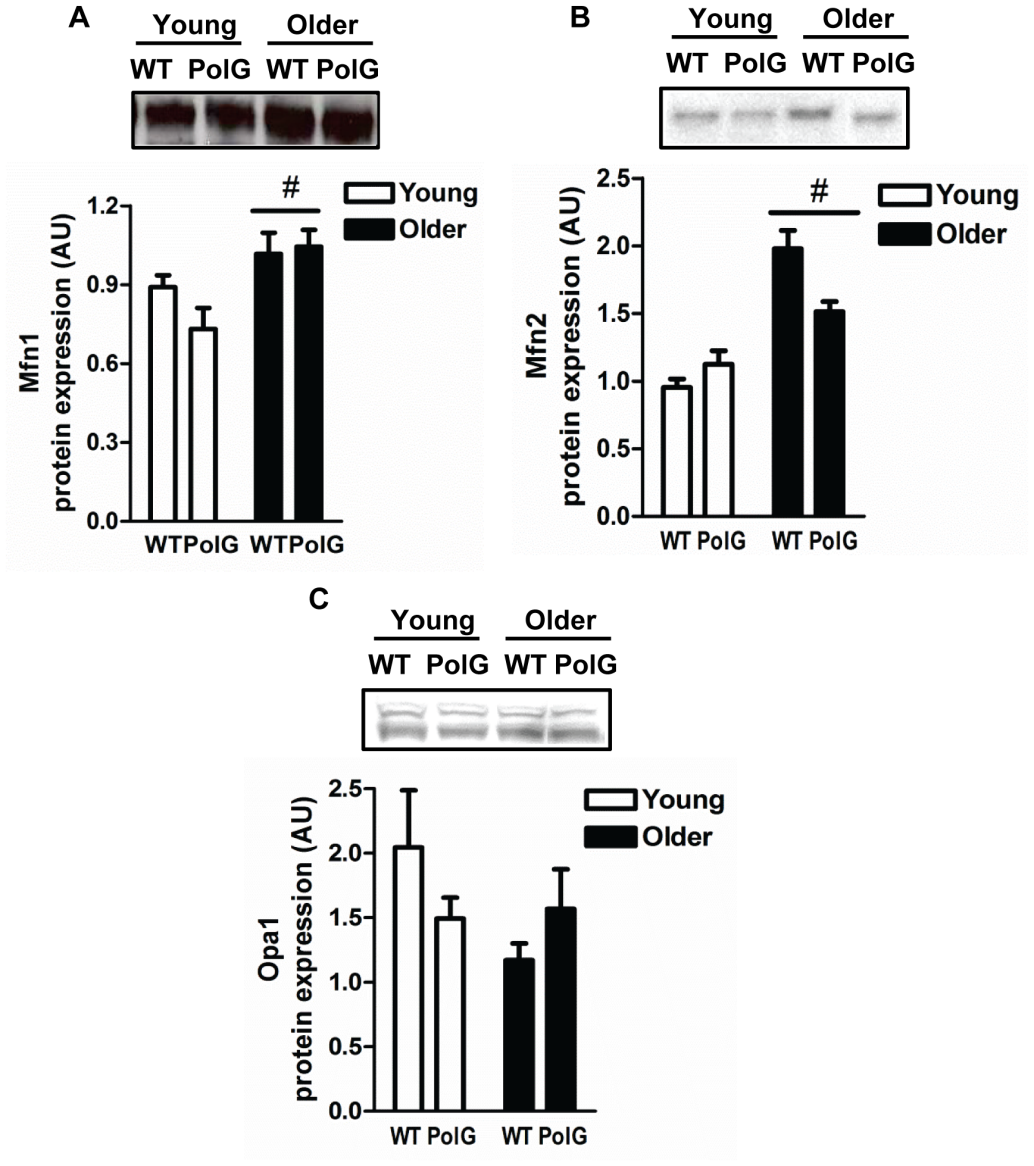
Altered mitochondrial fusion proteins in mtDNA mutator mice. Immunoblotting of mitochondrial fusion proteins Mfn1 (*A*), Mfn2 (*B*) and Opa1 (*C*) in skeletal muscle from young (3–6 mo) and older (8–15 mo) WT and PolG animals. Representative blots are shown above with a graphical summary of the data below (n = 7–13). Two bands were detected for Opa1 representing the long and short isoforms. Both bands were quantified and total Opa1 protein content displayed in the graph. Significance was set at P<0.05 and all data are represented as mean ± SE. Data are expressed as arbitrary units (AU). #P<0.05 main effect of age.

**Figure 4:**
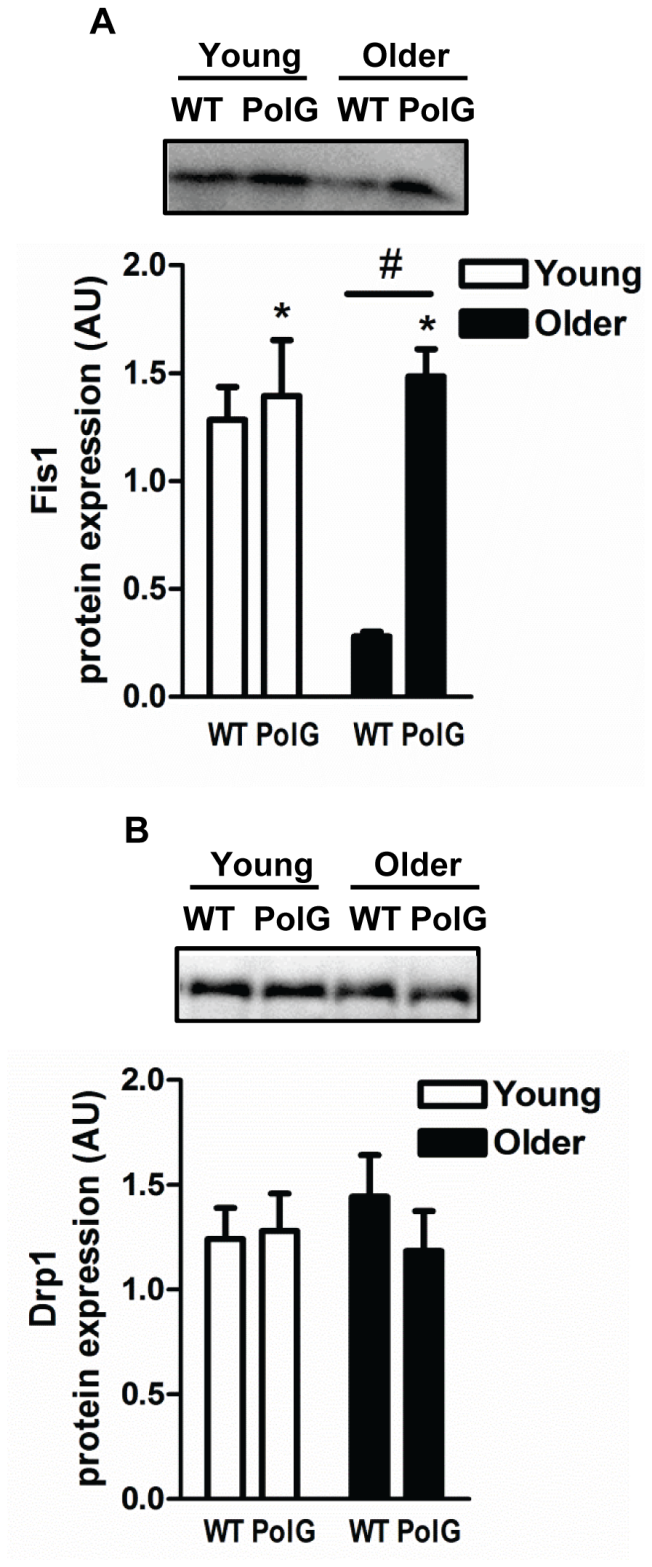
Altered expression of mitochondrial fission proteins in mtDNA mutator mice. Fis1 (*A*) and Drp1 (*B*) protein content was determined in muscle from young (3–6 mo) and older (8–15 mo) WT and PolG animals. A graphical summary along with representative blots are depicted (n = 7–13). Significance was set at P<0.05 and all data are represented as mean ± SE. Data are expressed as arbitrary units (AU). #P<0.05 main effect of age. *P<0.05 main effect of genotype.

To determine whether mitochondrial dynamics was altered in muscle from mtDNA mutator mice, we measured key fusion proteins mitofusin 1 and 2 (Mfn1 and Mfn2) and optic atrophy 1 (Opa1), and fission proteins, fission 1 protein (Fis1) and dynamin-related protein (Drp1). Both Mfn1 and Mfn2 levels were significantly higher with age (P<0.05; Fig. 3A and B), while Fis1 levels were reduced (P<0.05; Fig. 4A) in older animals when compared to young, indicating that fusion is upregulated during normal aging in muscle. In contrast, Fis1 levels were 5.3-fold higher (P<0.05) in muscle from older PolG animals when compared to age-matched WT (Fig. 4A). No changes were detected in Opa1 (Fig. 3C) or Drp1 (Fig. 4B) in any of the groups.

### Autophagy is not Impaired in mtDNA Mutator Mice

**Figure 5:**
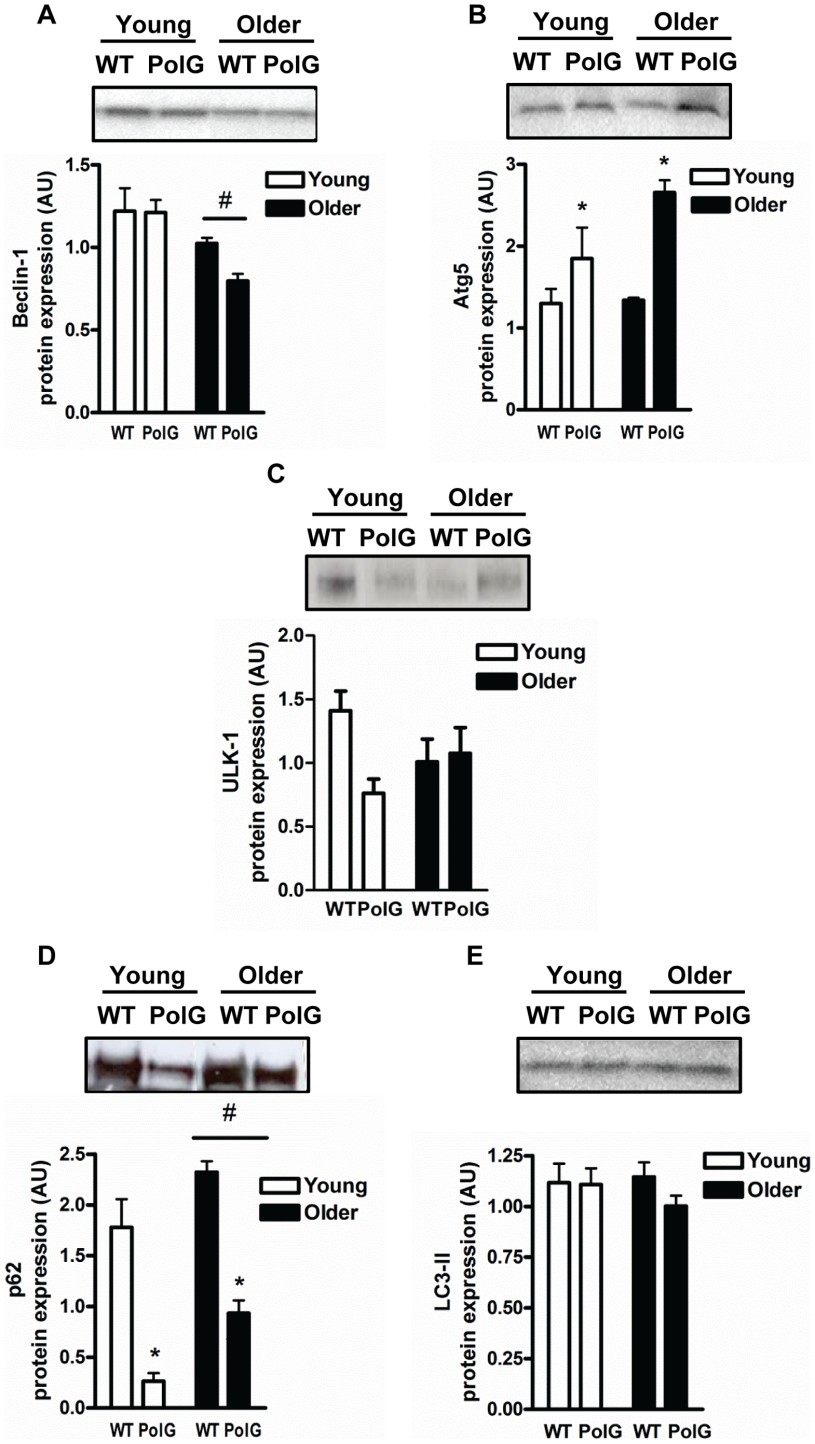
Autophagy proteins are upregulated in muscle from PolG animals. The content of autophagy proteins Beclin-1 (*A*), Atg5 (*B*), ULK1 (*C*), p62 (*D*), and LC3-II (*E*) was measured with Western Blotting in skeletal muscle from young (3–6 mo) and older (8–15 mo) WT and PolG animals. Representative blots are shown above with a graphical summary of the data below (n = 7–13). Significance was set at P<0.05 and all data are represented as mean ± SE. Data are expressed as arbitrary units (AU). #P<0.05 main effect of age. *P<0.05 main effect of genotype.

The regulation of mitochondrial turnover by autophagy occurs through lysosomal-mediated clearance of damaged organelles [1]. We found lower Beclin-1 content with age (P<0.05; Fig.5A), and this was more pronounced in animals with the PolG mutation. Additionally, muscle from older animals had an accumulation of the receptor cargo protein p62 when compared to their younger counterparts (P<0.05; Fig. 5D). Concomitantly, levels of the autophagy-related protein Atg5 (Fig. 5B), the membrane bound autophagosomal marker LC3-II (microtubule-associated protein 1 light chain 3) (Fig. 5E), and the mammalian homologue of Atg1, ULK1 (UNC-51-like kinase 1) (Fig. 5C) were not altered with normal aging. In contrast to normally-aging muscle, Atg5 content was higher (Fig. 5B) and p62 levels reduced (P<0.05; Fig. 5D) in PolG animals compared to WT suggesting greater autophagic degradation and activity in premature aging muscle.

### Changes in Mitochondrial Protein Import Machinery in Aging and PolG Animals

**Figure 6:**
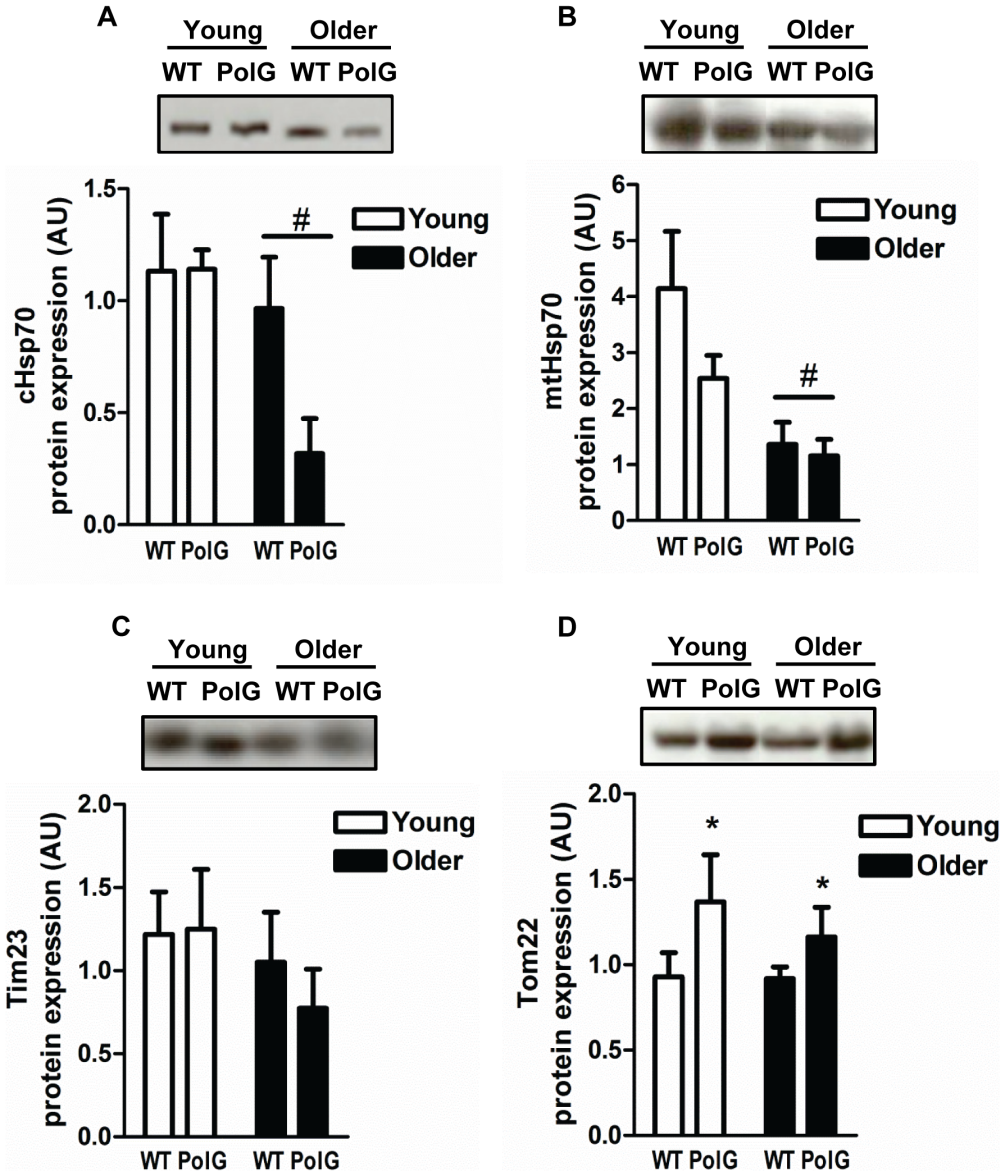
Effect of mtDNA mutations on mitochondrial protein import machinery. Levels of mitochondrial protein import machinery cytosolic Hsp70 (*A*), mitochondrial Hsp70 (*B*), Tim23 (*C*), and Tom22 (*D*) were measured in muscle from young (3–6 mo) and older (8–15 mo) WT and PolG animals. Representative blots are shown above with a graphical summary of the data below (n = 7–13). Significance was set at P<0.05 and all data are represented as mean ± SE. Data are expressed as arbitrary units (AU). #P<0.05 main effect of age. *P<0.05 main effect of genotype.

Mitochondrial protein import machinery plays a crucial role in mitochondrial biogenesis and turnover by regulating the import and translocation of hundreds of nuclear-encoded mitochondrial proteins into subcellular organelle compartments. Levels of the cytosolic and mitochondrial heat shock proteins (cHsp70 and mtHsp70, respectively) were reduced (P<0.05) in aging muscle (Fig. 6A and B) while no changes were observed in the translocase of the inner mitochondrial membrane 23 (Tim23; Fig. 6C) or the translocase of the outer mitochondrial membrane 22 (Tom22; Fig. 6D). Tom22 protein content was significantly increased in PolG animals when compared to WT and was the only mitochondrial import protein affected in these mutator mice (P<0.05; Fig. 6D).

## Discussion

Mutations and deletions in mtDNA have been reported in aging skeletal muscle [5], [6], [8], as well as in several types of muscle wasting conditions and myopathies in humans [26]. While a causal link between mtDNA mutations and sarcopenia has yet to be confirmed, there is strong evidence that mitochondrial dysfunction associated with greater mutation rates contribute to the muscle wasting process [27], [28]. Mitochondria have several intrinsic mechanisms to help defend against increasing damage, and we and others have proposed that dysregulation in these mitochondrial control processes play a prominent role in the skeletal muscle loss and atrophy observed in aging [25], [29]. To investigate these questions, we used the mtDNA mutator mouse with a mutation in the mtDNA proofreading enzyme PolG that results in a high mtDNA mutational load and a premature aging phenotype [9], [10]. Moreover, muscle from these animals display reduced endurance, weakness, and atrophy, which are in large part attributed to mitochondrial defects in ETC assembly and bioenergetics [12], [13], [30]. The current study shows for the first time that mtDNA mutations alter several mitochondrial quality control processes including biogenesis, fusion/fission, and autophagy, and that this is likely one of the underlying mechanisms contributing to sarcopenia in this model of premature aging. Importantly, these findings are also relevant to normal aging since mitochondrial mutation rates steadily increase in normally aged muscle. However, the responses observed in muscle from PolG mice differed from that of normally-aged muscle implying that the signals causing sarcopenia are not homogenous and provide support for a multifactorial etiology of muscle wasting.

The ability of skeletal muscle to adapt to cellular perturbations is highly dependent on mitochondrial regulation/biogenesis. Mitochondrial biogenesis involves the synthesis of new organelles from existing mitochondria and this process is predominantly regulated by PGC-1α [31]. Overexpression of this gene in skeletal muscle induces a fast- to slow- fiber type conversion and increases mitochondrial content and oxidative capacity through its modulation of a large group of genes involved in metabolism [32], [33]. Moreover, PGC-1α levels tend to be reduced in muscle wasting conditions including aging [24], [34], [35] and muscle-specific overexpression of PGC-1α has been shown to attenuate this muscle loss [36]. We found that muscle from older PolG animals had lower mtDNA content than WT animals and this was associated with normal COX (Complex IV) activity (Fig.1). Higher mtDNA mutations and deletions in mutator muscle affect the transcription and translation of mtDNA-encoded subunits leading to the reduced content of fully assembled ETC complexes [12]. As shown in our study, the specific activities of these complexes (i.e. COX) are not altered suggesting that the remaining assembled holoenzyme complexes are functional [12]. Despite the lower mtDNA content, mitochondrial biogenesis proteins appeared to be upregulated in muscle from PolG animals as indicated by the higher PGC-1α, NRF-1, and Tfam content (Fig. 2). Changes in PGC-1α were not associated with greater activation of AMPKα, which has been shown to phosphorylate PGC-1α, increase its transcriptional activity, and the expression of its downstream target genes [37]. Our findings differ from previous reports showing lower levels of mitochondrial biogenesis regulators in these mice [13], [38]. The divergent response in our results may be due to differences in age, as well as the extent of mtDNA mutation loads in the animals. While mutation rates in these mutator mice have been described previously [10], we did not directly measure this in our study. Nonetheless, similar compensatory increases in factors stimulating mitochondrial biogenesis have been observed in other experimental models of mitochondrial dysfunction [36], [39]. A recent study by Moraes group [40] showed that muscle-specific overexpression of PGC-1α in PolG animals led to improvements in skeletal muscle function. Moreover, it was recently reported that endurance training in these animals induces mitochondrial biogenesis and improvements in overall muscle function [38]. Collectively, these studies indicate that there may be mitochondrial-nuclear crosstalk pathways, whereby increased mitochondrial damage and deficits in oxidative capacity produce a signal that is then recognized by the nucleus to induce mitochondrial biogenesis factors that can attenuate further cellular damage. More work elucidating the potential involvement of mitochondrial-nuclear retrograde signaling pathways in these compensatory responses in PolG muscle is certainly required as these are relatively unknown at present.

It is now well established that mitochondria are not static but rather dynamic organelles capable of altering their shape and size in response to ever changing cellular demands. Mitochondrial fusion and fission events not only dictate the morphology of the organelle but are also responsible for maintaining mitochondrial turnover through their contributions to the synthesis (biogenesis) and degradation of organelles [14]. Mitochondrial fusion is primarily regulated by members of the dynamin-related GTPases, mitofusin 1 and 2 (Mfn1 and Mfn2) and optic atrophy protein 1 (Opa1) that tether and fuse the outer and inner mitochondrial membranes, respectively [41], [42]. In contrast, fission proteins such as the dynamin-related protein 1 (Drp1) and fission protein 1 (Fis1) are responsible for the division of mitochondria [43], [44]. It is well established that mitochondrial morphology is altered in aging skeletal muscle. These mitochondria appear enlarged, highly interconnected, and have reduced mitochondrial function [45]–[47] indicating that aging may cause a shift towards greater mitochondrial fusion events [48]. In our study, higher Mfn1 and Mfn2, and lower Fis1 levels (Fig. 3 and Fig. 4) were observed in muscle from normally-aging animals, a finding that is consistent with others [49]. Higher levels of fusion in aged muscle is likely a compensatory response by the cell to dilute excessive levels of damaged organelles through fusion of neighboring “healthy” non-damaged mitochondria and the mixing of their contents [50]. Consistent with this hypothesis, animals with muscle-specific deletions of Mfn1 and Mfn2 have lower muscle mass associated with reduced mtDNA content, higher mutation rates, and overall mitochondrial dysfunction [51]. Additionally, disruption of Mfn1 in mtDNA mutator mice exacerbates mitochondrial defects and induces lethality, confirming the importance of mitochondrial fusion in the maintenance of mtDNA integrity and its protective role against pathogenic mtDNA mutations [51].

Excessive amounts of fission and autophagy have been proposed to precede muscle atrophy in experimental models of muscle disuse (i.e. denervation and fasting) [29]. This has been confirmed by experimentally altering the levels of key fission regulators Drp1 and Fis1, or autophagy proteins such as BNIP3 (Bcl2/adenovirus E1B interacting protein 3). Specifically, overexpression of these proteins leads to mitochondrial fragmentation, reduced bioenergetics, higher autophagy levels, and muscle atrophy, while repression of these proteins blocks mitochondrial fission and prevents acute muscle wasting [29]. Fission regulates autophagy by providing the substrates required for lysosomal degradation. During biogenesis, fission identifies damaged mitochondria and targets them for autophagic degradation which prevents their re-entry into the mitochondrial reticulum [15]. Consistent with previous experimental models of muscle wasting, fission and autophagy levels were upregulated in PolG animals, as indicated by higher levels of Fis1 and Atg5, and lower p62 (also named sequestosome 1; SQSTM1) content (Fig. 5). The lower levels of p62 associated with enhanced autophagy may initially be considered counterintuitive since p62 links ubiquitinated proteins to autophagosomes. However, p62 is rapidly degraded during autophagy and therefore lower p62 content is typically associated with higher autophagy activity [52], [53]. P62 also binds to LC3-II which is considered a robust autophagosomal marker of lysosomal turnover [54]. Levels of LC3-II were unchanged in mtDNA mutator mice and similar unparalleled responses between LC3-II and p62 have previously been reported in skeletal muscle [49]. Additionally, levels of the ULK1 protein kinase, a key factor in the initiation of the autophagy process were not altered in any of the groups. Recent studies have demonstrated that during conditions of nutrient deprivation, ULK1 is directly phosphorylated and activated by AMPK to induce autophagy [55], [56]. Overall, our data is in line with electron microscopy experiments showing abnormal mitochondrial morphology including fragmented cristae and the presence of vacuoles in muscle from PolG mice [38]. Higher fission observed in muscle from older PolG mice is also consistent with previously published data by our group showing greater apoptosis in these animals [9], [12]. Based on these findings, we propose that dysregulation in mitochondrial control processes mediated by higher fission and autophagy markers contribute to the premature muscle loss observed in mtDNA mutator mice.

In normal-aging muscle, autophagy proteins were downregulated and this is consistent with previous observations by our group and others showing that autophagy is impaired in aging muscle [25], [57], [58]. In addition to lower autophagy-related proteins, aged muscle cells also display an accumulation of intracellular debris such as lipofuscin, a common indicator of impaired lysosomal function and the final step in the autophagy process [59], [60]. Despite these findings, not all studies demonstrate impaired autophagy with some showing higher levels of autophagy proteins within muscle. For example, O’Leary *et al.* recently reported greater levels of autophagy-related machinery Atg7 and Parkin in muscle from 35-month old Fischer 344×Brown Norway rats [49]. The divergent results between these studies can be attributed to a number of factors including the age of animals, as well as differences in muscle fiber types being studied. It is noteworthy to mention that while we would have liked to directly measure autophagic flux in our tissue preparations, methods permitting this are currently limited. Therefore, similar to other studies, we assessed autophagy by measuring a number of key robust autophagy markers that are tightly associated with changes in autophagic flux and activity.

Finally, we investigated the effect of mtDNA mutations on the mitochondrial protein import machinery (PIM). Since the majority of mitochondrial proteins required for biogenesis are encoded in the nucleus, a specialized group of machinery composed of chaperones and translocases of the outer and inner membrane (TOMs and TIMS, respectively) exists to facilitate their import into the organelle [31]. Moreover, the PIM acts as a metabolic sensor adapting to perturbations in cellular energy by modifying the rate of precursor protein import into subcellular compartments [61], [62]. Precursor protein import is upregulated in response to muscle use [62], as well as with mtDNA depletion, mitochondrial disease, and aging and these compensatory increases are mediated, in part, by changes in the levels of PIM components [24], [63], [64]. In our study, we found reduced levels of important chaperone proteins in normally-aged muscle. Tom22 content, however, was markedly higher in PolG animals when compared to control (Fig. 6). This has significant implications since Tom22 is an essential protein import factor required for the proper assembly and import of Tom40, the primary component of the translocase of the outer mitochondrial membrane (TOM) complex from which the majority of proteins enter mitochondria [65]. In fact, overexpression of Tom22 in HeLa cells leads to the greater assembly of Tom40 into the TOM complex coincident with higher protein import rates [65], [66]. Interestingly, the expression of several of the PIM components is driven by NRF-1 and NRF-2 [67]–[69] establishing a link between muscle plasticity, biogenesis, and protein import adaptations in premature aging. Thus, we demonstrate for the first time the adaptability of the mitochondrial PIM in mtDNA mutator mice and identify additional molecular pathways that may contribute to muscle wasting in premature aging.

In summary, data from this study provides further evidence for the importance of mitochondrial control processes in muscle atrophy. Moreover, although higher mtDNA mutations are central to both the etiology of muscle loss in mtDNA mutator mice and normal-aging mice, the protein expression pattern in these conditions appears to be quite distinct (Table 1). The divergent response in muscle between these two models of atrophy highlights the diversity, as well as complexity in the underlying signals involved in the pathology of muscle wasting. Nonetheless, investigating the consequences of accelerated mtDNA mutation rates in progeroid aging models such as the mtDNA mutator mouse will help us to better understand the factors regulating muscle quality in aging and in a large number of diseases affected by muscle wasting. Thus, not only do these studies help identify potentially novel therapeutic targets to protect against muscle wasting but they also allow the opportunity for the development of new models in the study of aging.

**Table 1:** Comparison of changes in mitochondrial quality control proteins in premature and normally-aging mice.

	Premature Aging (PolG)	Normal Aging (WT)
***Biogenesis***		
AMPKα activation	**↔**	**↓**
PGC-1α	**↑**	**↔**
NRF-1	**↑**	**↔**
Tfam	**↑**	**↔**
***Fusion***		
Mfn1	**↔**	**↑**
Mfn2	**↔**	**↑**
Opa1	**↔**	**↔**
***Fission***		
Fis1	**↑**	**↓**
Drp1	**↔**	**↔**
***Autophagy***		
Beclin-1	**↔**	**↓**
Atg5	**↑**	**↔**
ULK1	**↔**	**↔**
P62	**↓**	**↑**
LC3II	**↔**	**↔**
***Protein import***		
cHsp70	**↔**	**↓**
mtHsp70	**↔**	**↓**
Tim23	**↔**	**↔**
Tom22	**↑**	**↔**

Changes are expressed relative to appropriate controls. Premature aging (PolG vs. age-matched WT) and normal aging (young WT 3–6 mo vs. older WT 8–15 mo).
